# Syringomyelia Resembling Leprosy

**Published:** 1909-03-20

**Authors:** 


### MEDICINE.

### SYRINGOMYELIA RESEMBLING LEPROSY.

There is a variety of syringomyelia in which the main symptoms consist in the recurrence of apparently causeless ulcers, whitlows, and sore places on the hands or feet or other parts. Before it was discovered that these sores are due to traumata that pass unrecognised by the patient on account of his loss of power to distinguish heat from cold and pain from touch, they were regarded as a special skin disease, and descriptions of them will be found in dermatological papers and books under the heading of Morvan's disease. We now know that this is a manifestation of syringomyelia.

It will be remembered that there is a special variety of leprosy in which peripheral inflammation of the sensory nerves gives rise to varying degrees of cutaneous insensibility, whilst indolent ulcers also arise in the affected parts. Seeing, therefore, that sensory disturbances and cutaneous sores may be the chief features both of syringomyelia and of leprosy, it is not surprising that there are patients in whom it is a matter of the very greatest difficulty to decide whether their lesions are due to leprosy or to syringomyelia. The following example is recorded by M. Alex Renault:—

The patient was a man aged 52, a commercial traveller, who had been abroad a good deal. There was nothing to note in the family history. As regards the personal history, the man had been quite well till at eighteen he contracted syphilis. The chancre was typical, but there were few secondary symptoms. Mercurial treatment was thorough whilst it lasted, but the patient stopped it two months after the last trace of the chancre had gone.

No further trouble occurred until he was thirty-two. At this time he had been for three years in Borneo, where he had undoubtedly exceeded both as regards tobacco and spirituous liquors. It was then that he noticed multiple tumours developing both on his arms and on his legs. Some of the lumps soon ulcerated, and at that time they were regarded as undoubtedly syphilitic gummata. They healed slowly, leaving scars that were depressed, thin-skinned, smooth, and with a ring of pigmentation around each, and were in fact typical of syphilis. The last of these ulcers did not finally close up until he was forty, eight years after they first came, and this notwithstanding the continued use of iodide of potassium.

When the patient was forty-seven definite paresis of both hands and both feet set in. The patient had the greatest difficulty in recognising objects by the sense of touch. He could scarcely button up his trousers or write with a pen. The skin of his limbs was found to be insensitive.

A little more than a year later the dorsal surface of his hands became covered with bullæ, which were followed by ulceration. Several physicians having seen him in hospital, the conclusion come to was that the insensibility and the trophic changes pointed to leprosy. Iodide of potassium and chaulmoogra oil were prescribed, and the indolent ulcers were touched here and there with the cautery.

Notwithstanding the persistence of paræsthesia, paresis, and recurrent ulcerations of the hands and feet—but not of other parts—the man managed to superintend certain light work for a time. The ulcers that kept coming were in themselves painful on walking, although they occurred in comparatively insensitive skin. A year later the trouble was so bad that the man was once more laid up in hospital, the diagnosis by various physicians wavering between leprosy, syringomyelia, and tertiary syphilis. Some relief followed the administration of 45 grains of potassium iodide daily for some months, and the man was able to return to light work, although the latter entailed a good deal of walking. A little over a year later, however, he was in a worse plight than ever, with ulcerations of his fingers, hands, toes, and feet, perforating ulcers of the soles, and loss of a phalanx of his little toe. Notwithstanding all treatment these lesions could not be cured, and they were as bad as ever at fifty-two.

At that time all four limbs were paræsthetic throughout their length. On careful analysis it could be determined that the sense of touch was very obtuse, but not absolutely lost, though pressure upon the skin had to be considerable before it was perceived. Ability to distinguish heat, cold, and painful stimuli was quite gone. Sensibility upon the head, face, neck, chest, abdomen, and back was quite normal. The knee-jerks were present, and if anything relatively brisk. There was a decided tendency to ankle clonus. The plantar reflexes were flexor. There was a marked atrophy of the muscles of the thenar eminences, and the interossei of the fourth left space were defective in power as shown by the fact that the ring-finger could not be fully extended, nor could the little finger be properly approximated to it in the extended position. The right foot was also much deformed. The big toe was in a position of extreme flexion, and the other toes were, so to speak, crumpled together; the fifth was reduced to a mere stump owing to suppuration and the loss of the second phalanx. On the sole of the foot near the toes there were two perforating ulcers, the one corresponding to the head of the first metatarsal bone, the other to the head of the fifth. The median nerve on either side was thickened without being nodular. The viscera all seemed healthy; there was no albuminuria.

It may seem simple enough, at any rate upon paper, to attribute the whole of the above patient's symptoms and lesions to tertiary syphilis; but it was the opinion of those who saw him that the diagnosis lay between syringomyelia and leprosy and not syphilis. In any case, there are patients with similar lesions in whom syphilis can be absolutely excluded, and the whole difficulty is to decide whether the mischief is due to syringomyelia or to leprosy. As M. Renault points out, it is sometimes quite impossible to distinguish between them.

In each of the two maladies in question three orders of symptoms are met with, namely, sensory disturbances, trophic changes or skin lesions, and reflex disorders. As regards the sensory disturbances, Jean Selme has said that in leprosy the loss of sensibility is symmetrical and is very prone to affect the four limbs, leaving the face and trunk free as a rule; the maximum anæsthesia is superficial, decreases as greater depths are stimulated, and is also greater at the distal ends of the limbs than it is at the levels of their junctions with the body. Moreover, it often exhibits some degree of "dissociation—*i.e.* there may be absolute analgesia and inability to detect hot or cold stimuli, and yet stimuli of mere contact may still be vaguely perceived.

In syringomyelia the dissociation of the sensory abnormalities is usually much more perfect—cutaneous sensibility may be undiminished, although there may be absolute analgesia and thermo-anæsthesia. Besides this the trunk may be involved as well as or independently of the limbs; and instead of the analgesic and thermo-anæsthetic areas gradually shading off into normal, there is often an absolutely sharp line of demarcation.

In M. Renault's patient sensibility was natural upon the head and trunk, although the four limbs were completely anæsthetic to painful and thermal stimuli, and nearly so to those of simple touch. If the sensory disturbances alone were relied upon the case was clearly one of leprosy.

Turning now to the reflexes, in leprosy the knee-jerk tends to be diminished and there is no ankle clonus. In certain cases of syringomyelia, on the other hand, the knee-jerks are apt to be increased and associated with ankle clonus. Upon this ground one would diagnose syringomyelia.

Next, as regards the trophic changes, the patient exhibited decided atrophy of the thenar muscles of each hand, and of one fourth palmar interosseous muscle. There were the bullous and ulcerating lesions that recurred for months and months upon the hands and feet. There were perforating ulcers upon the sole of one foot. Suppurative dactylitis had caused partial loss of a little toe. None of these points help greatly in the differential diagnosis, for absolutely identical lesions may occur both in syringomyelia and in leprosy.

Are there any other points that might assist one? There are two, namely, the state of the nerves, and the results of bacteriological examinations. Both the median nerves in the above case were obviously thickened; each was uniform and about the size of a goose quill. This argues fairly strongly in favour of leprosy, for in a case suspected to be the anæsthetic type of the latter, one of the first things one does is to feel for this thickening of the affected nerves, and it is by no means usual to find similar thickening in syringomyelia.

On the other hand, all attempts to discover the bacilli of leprosy proved abortive. It is true that leprosy bacilli may sometimes be remarkably localised, as in a case recorded by Souza-Martins, of Lisbon, who found in a patient, dead of what appeared to be syringomyelia, a cavity full of characteristic leprosy bacilli in the cervical region of the spinal cord. Nevertheless, one usually expects to find the bacilli in the blood, in the fluid in the bullæ, in the discharge from the sores, in the nasal mucus, in the saliva, or in the sero-purulent fluid coming from the perforating ulcers.

The fact that some of the sores healed completely, if only very slowly, under local treatment, together with the administration of iodide of potassium, might be regarded as strong evidence in favour of syphilis and against leprosy, and neither for nor against syringomyelia; but upon the other hand, the facts that there were no other syphilitic lesions upon the body, and that the sores finally resisted anti-syphilitic treatment altogether would argue against syphilis. The diagnosis must remain obscure, for the patient is still alive.

